# Knockout of Eva1a leads to rapid development of heart failure by impairing autophagy

**DOI:** 10.1038/cddis.2017.17

**Published:** 2017-02-02

**Authors:** Shu Zhang, Xin Lin, Ge Li, Xue Shen, Di Niu, Guang Lu, Xin Fu, Yingyu Chen, Ming Cui, Yun Bai

**Affiliations:** 1 Department of Cardiology, Peking University Third Hospital, Beijing 100191, China; 2 Department of Immunology, School of Basic Medical Sciences, Peking University Health Science Center, Beijing 100191, China; 3 Peking University Center for Human Disease Genomics, Beijing 100191, China; 4 Department of Cell Biology, School of Basic Medical Sciences, Peking University Health Science Center, Beijing 100191, China; 5 Department of Physiology, Yong Loo Lin School of Medicine, National University of Singapore, Singapore 117597

## Abstract

EVA1A (Eva-1 homologue A) is a novel lysosome and endoplasmic reticulum-associated protein that can regulate cell autophagy and apoptosis. Eva1a is expressed in the myocardium, but its function in myocytes has not yet been investigated. Therefore, we generated inducible, cardiomyocyte-specific *Eva1a* knockout mice with an aim to determine the role of *Eva1a* in cardiac remodelling in the adult heart. Data from experiments showed that loss of *Eva1a* in the adult heart increased cardiac fibrosis, promoted cardiac hypertrophy, and led to cardiomyopathy and death. Further investigation suggested that this effect was associated with impaired autophagy and increased apoptosis in *Eva1a* knockout hearts. Moreover, knockout of *Eva1a* activated Mtor signalling and the subsequent inhibition of autophagy. In addition, *Eva1a* knockout hearts showed disorganized sarcomere structure and mitochondrial misalignment and aggregation, leading to the lack of ATP generation. Collectively, these data demonstrated that Eva1a improves cardiac function and inhibits cardiac hypertrophy and fibrosis by increasing autophagy. In conclusion, our results demonstrated that Eva1a may have an important role in maintaining cardiac homeostasis.

Cardiac remodelling is a pivotal pathological phenomenon that occurs during the clinical course of stress-induced heart failure and represents an independent risk factor for subsequent cardiac morbidity and mortality.^1^ Specifically, cardiac hypertrophy is characterized by an abnormal enlargement of the heart muscle as a result of the increased myocyte cell size and abnormal proliferation of non-muscle cells.^2, 3^ Cardiac fibrosis is characterized by excessive extracellular matrix accumulation and fibroblast deposition, which eventually destroys organ architecture and abolishes normal function.^4, 5, 6^ Cardiac remodelling is a major biological determinant of fatal events, including heart failure, severe arrhythmias, and sudden cardiac death.^7, 8^ Thus, elucidating the mechanisms implicated in cardiac protection against remodelling is of great significance.

Autophagy is a highly conserved catabolic process that is involved in delivering cytoplasmic components to lysosomes for degradation. It has a pivotal role in maintaining the cellular environment of the heart.^9^ Effective autophagy in cardiomyocytes is necessary for normal metabolism and cellular survival. The inability of autophagy to completely remove damaged structures results in a progressive accumulation of cellular debris, including cytosolic protein aggregates and defective mitochondria. Previous studies have demonstrated that dysregulation of autophagy can promote the development of many forms of heart disease as well as cardiac remodelling.^10, 11, 12, 13, 14^ Moreover, autophagy is inhibited during the progression of cardiac hypertrophy, which is an important part of cardiac remodelling.^9^ Studies have found that facilitation of autophagy can attenuate cardiac remodelling.^15, 16^ However, the precise role of autophagy in cardiac remodelling remains to be elucidated.

The Eva-1 homologue A (EVA1A; also known as transmembrane protein 166 [TMEM166] and family with sequence similarity 176 [FAM176A]) is a novel lysosome and endoplasmic reticulum-associated protein that can regulate cell autophagy and apoptosis.^17, 18^ It is conserved in humans, chimpanzees, rats, mice, and dogs, indicating that it may have important functions in vertebrates. Previous studies have shown that EVA1A is expressed in a cell-type- and tissue-type-specific manner, and is significantly downregulated in cancer tissues.^18, 19, 20^ Furthermore, *in vivo* and *in vitro* experiments have demonstrated that EVA1A overexpression inhibits tumour cell proliferation by both autophagy and apoptosis.^19, 20^ Latest research shows that *Eva1a* deletion impairs the generation of newborn neurons by activating the PIK3CA–AKT axis and inhibiting autophagy.^21^ However, its role in cardiac remodelling remains to be elucidated.

Therefore, we aimed to determine the role of *Eva1a* in cardiac remodelling in the adult heart. To this end, we generated an inducible myocyte-specific *Eva1a* knockout mouse model to investigate the role of Eva1a in the adult heart.

## Results

### Generation of tissue-specific *Eva1a* knockout mice

**Figure 1:**
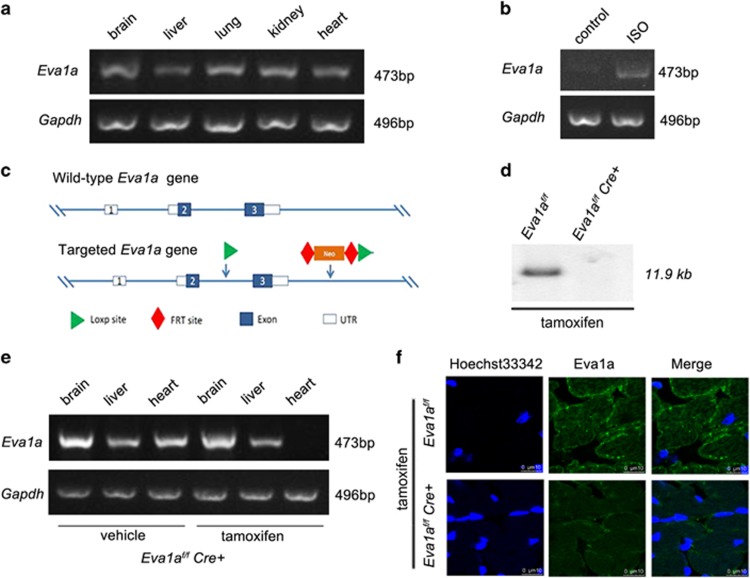
Characterization of *Eva1a* expression in *Eva1a*^*f/f*^ and *Eva1a*-deficient mice. (**a**) Representative RT-PCR results showing endogenous *Eva1a* expression in different tissues from wild-type mice. (**b**) Representative RT-PCR results showing endogenous *Eva1a* expression in ISO-induced cardiac remodelling. (**c**) Scheme to generate *Eva1a*-deficient mice. (**d**) Southern blot analysis to detect *Eva1a* expression in the heart in *Eva1a*^*f/f*^ and *Eva1a*-deficient mice. (**e**) Representative RT-PCR results showing endogenous *Eva1a* expression in different tissues from *Eva1a*^*f/f*^ and *Eva1a*-deficient mice. (**f**) Eva1a expression was detected by immunofluorescence assay

Consistent with previous reports, RT-PCR results showed that *Eva1a* mRNA was moderately expressed in adult heart tissue (Figure 1a). To determine whether Eva1a is involved in cardiac remodelling, we examined the gene expression in heart extracts from adult mice after they were treated with isoproterenol (ISO) for 2 weeks. As shown in Figure 1b, there was a significant increase in *Eva1a* expression in ISO-induced hypertrophied hearts, indicating its potential involvement in cardiac remodelling. To determine the role of Eva1a in cardiac remodelling, we generated temporally controlled cardiac-specific *Eva1a*-deficient mice. *Eva1a*^*flox/flox*^ mice had two *LoxP* sequences flanking exon 3 of the mouse *Eva1a* gene and a neo cassette. *Cre*-mediated deletion led to a deletion mutation because of direct splicing from exon 3 and the neo cassette, producing a small truncated non-functional peptide (Figure 1c). Southern blot analysis was used to confirm the deletion of *Eva1a* in heart tissue by a tamoxifen-inducible *Cre* recombinase (*MerCreMer*; Figure 1d). The resulting *Eva1a*^*flox/flox*^*:α-MHC MerCreMer*^*+*^ (*Eva1a*^*f/f*^*Cre*^*+*^) mice were indistinguishable in appearance from their age-matched control *Eva1a*^*flox/flox*^*:α-MHC MerCreMer*^*−*^ (Eva1a^f/f^) littermates. In the *Eva1a*^*f/f*^*Cre*^*+*^ mice that had been treated with tamoxifen for 3 days, *Eva1a*-deficient mice were identified by RT-PCR and immunofluorescence analyses, and *Eva1a* gene and protein expression levels were significantly reduced in the heart of *Eva1a*^*f/f*^*Cre*^*+*^ mice (Figures 1e and f).

### Loss of *Eva1a* leads to rapid early mortality and contractile dysfunction

**Figure 2:**
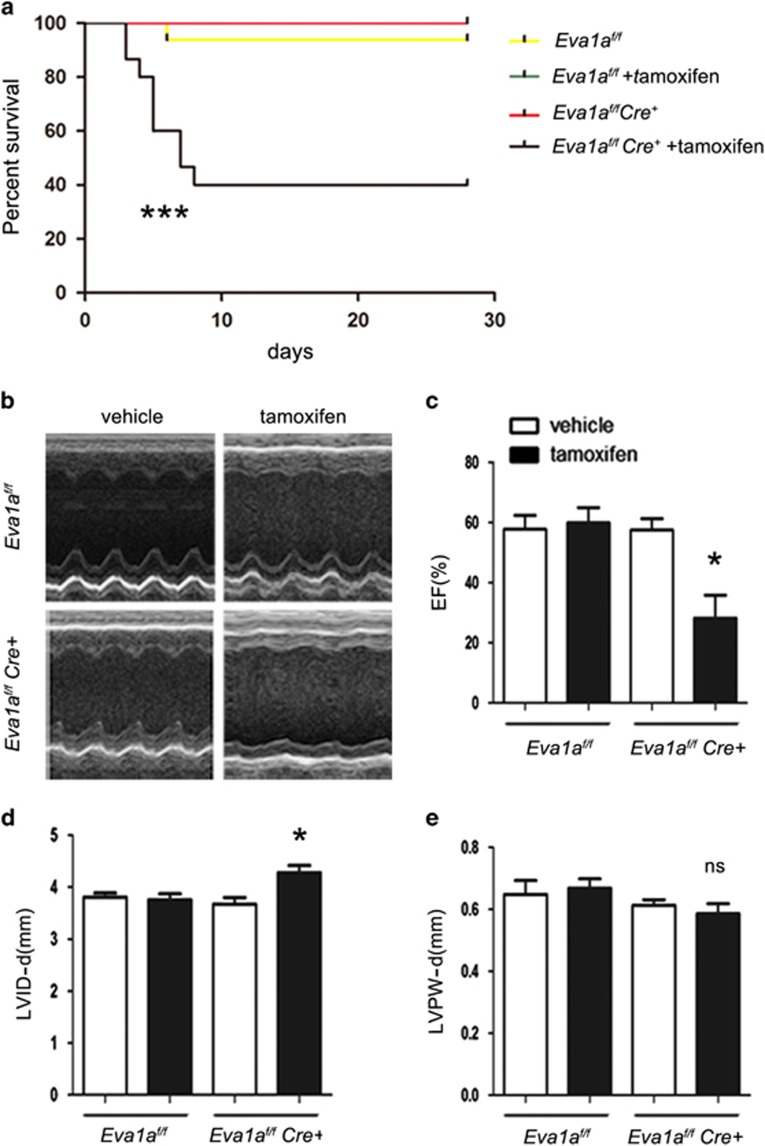
Effects of *Eva1a*-deficiency on cardiac function and structure. (**a**) Kaplan–Meyer survival curve (***P<0.0001, *n*=10). (**b**) Representative M-mode echocardiography images showing left ventricular wall thickness and systolic function in different mice. (**c**) Ejection fraction (EF) was significantly lower in *Eva1a*-deficient mice than in the other mice (*P<0.05, *n*=8). (**d**) Echocardiographic analysis revealed enlarged left ventricular diastolic dimension (LVID-d) was significantly higher in *Eva1a*-deficient mice than in the other mice (*P<0.05, *n*=8). (**e**) Diastolic left ventricular wall thickness (LVPW-d) did not significantly differ between *Eva1a*-deficient mice and the other groups (*n*=8)

Comparison of the survival rates between *Eva1a-*ko mice and their littermates with the *Eva1a*^*f/f*^ genotype revealed a significant difference between these two groups. *Eva1a-*ko mice that were treated with tamoxifen for 3 days started to die, and fewer than half of the mice survived to 28 days (Figure 2a).

**Figure 3:**
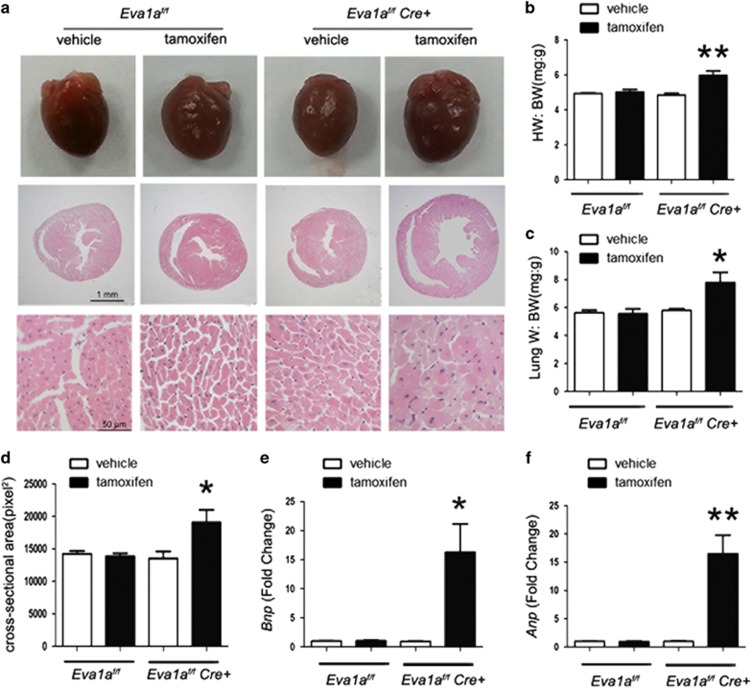
Effects of *Eva1a* deficiency on cardiac hypertrophy. (**a**) The hearts of *Eva1a* deficient mice were enlarged on gross morphology. Haematoxylin and eosin (H&E) staining of heart sections. Scale bar=1 mm or 50 *μ*m. (**b**) The ratio of heart weight to body weight (HW/BW) did not significantly differ between *Eva1a*-deficient mice and the other mice (**P<0.01, *n*=9). (**c**) The ratio of lung weight to body weight (LW/BW) was significantly different between *Eva1a*-deficient mice and the other mice (*P<0.05, *n*=9). (**d**) Measurements of two-dimensional cardiomyocyte cross-sectional areas (*P<0.05, *n*=4). (**e** and **f**) Analysis of hypertrophy markers *Bnp* (**e**) and *Anp* (**f**) by qRT-PCR (*P<0.05, **P<0.01, *n*=5)

Echocardiography was performed to determine alterations in the cardiac structure and function of *Eva1a-*ko mice. The left ventricular end-diastolic diameter (LVID-d) and left ventricular end-systolic diameter (LVID-s) were both higher in the *Eva1a* knockout mice than in the other groups (Figures 2b and d). Left ventricular function was severely impaired in *Eva1a* knockout mice as indicated by a significant decline in percent fractional shortening (FS) and a significant decrease in the ejection fraction (EF; Figures 2b and c), whereas the FS and EF values in the other groups showed no marked differences. In addition, the diastolic left ventricular wall thickness (LVPW-d) did not significantly differ between *Eva1a* knockout mice and the other groups (Figures 2b and e). Consistent with these findings, dissection of anaesthetised moribund *Eva1a*-deficient mice showed obvious pulmonary oedema, and the lung weight normalized to the body weight was significantly higher in the *Eva1a*-deficient mice than in the other groups (Figure 3c). The mice were analysed for several cardiac remodelling-related indicators at 1 day before tamoxifen injection and at 3, 7, and 14 days post tamoxifen injection. At 3 days post tamoxifen injection, the LVID-d was greater in the *Eva1a*-deficient group than in the other groups; there was also a marked difference at 14 days (Figure 2d). In addition, the EF and FS values were significantly lower in the *Eva1a*-deficient group than in the other groups from 3 days after the injection (Figure 2c). Collectively, these results indicate that *Eva1a*-deficient mice displayed worse protection of cardiac function than the other mice.

### Knockout of *Eva1a* promotes cardiac hypertrophy

Cardiac hypertrophy is a major predictor for the development of cardiac remodelling. We carried out pathological analysis to investigate the role of *Eva1a* in cardiac hypertrophy. As shown in Figures 3a and b, the heart weight/body weight (HW/BW) ratio was dramatically higher in the *Eva1a*-deficient mice than in the other groups (Figures 3a and b). In addition, the cross-sectional cardiomyocyte area was higher in the *Eva1a*-deficient mice than in the other groups. However, there was no significant difference between the groups expressing *Eva1a* (Figures 3a and d). The expression of the hypertrophic markers *Anp* and *Bnp* were significantly increased in the *Eva1a* knockout group, but the *β-Mhc* expression was not increased (Figures 3e and f). Taken together, these results indicated that Eva1a may exert a beneficial effect on cardiac hypertrophy.

### *Eva1a* knockout increases cardiac fibrosis *in vivo*

**Figure 4:**
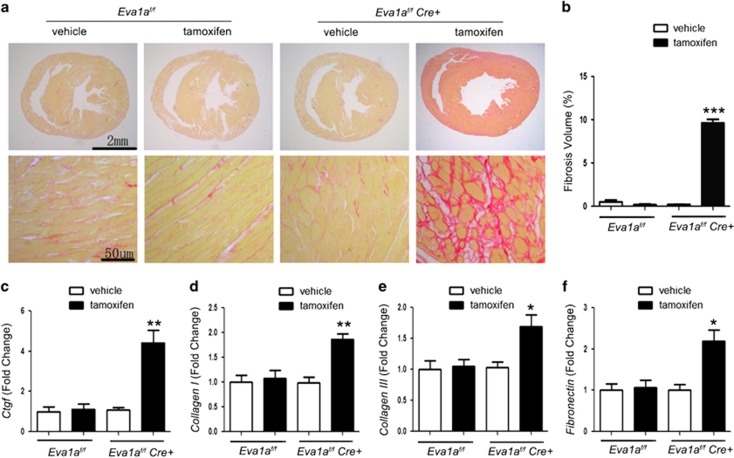
Effects of *Eva1a* deficiency on cardiac fibrosis. (**a**) Representative micrographs of picrosirius red-stained sections of the ventricle. Red areas represent collagen. Scale bar=2 mm or 50 *μ*m. (**b**) Quantification of cardiac interstitial collagen content in picrosirius red-stained sections. Results are expressed as the ratio of collagen area to heart area (***P<0.001, *n*=4). (**c**–**f**) Analysis of fibrosis markers *Ctgf* (**c**), *Collagen I* (**d**), *Collagen III* (**e**), and *Fibronectin* (**f**) by qRT-PCR (*P<0.05, **P<0.01, *n*=6)

Cardiac fibrosis is an important process in pathological cardiac remodelling and can lead to heart failure. Therefore, we investigated the effect of Eva1a on cardiac fibrosis by Sirius red staining. At 3 days post tamoxifen injection, collagen accumulation was absent in all the groups (data not shown); however, collagen deposition was increased in the *Eva1a*-ko heart tissues at 2 weeks after injection (Figure 4a), whereas collagen accumulation was absent in the other groups (Figure 4a). Results of the quantitative analysis revealed that the collagen volume in the myocardium was lower in the other groups than in the *Eva1a*-deficient group (Figure 4b). There was decreased fibrosis in the groups with *Eva1a*; this may be because of the increased collagen degradation or decreased collagen synthesis in response to tissue damage. Fibrosis is characterized by excessive accumulation of collagen and other extracellular matrix components. Development of the fibrotic extracellular matrix is driven by connective tissue growth factor (CTGF) and other cytokines and growth factors, with overload of factors such as collagen I, collagen III, fibronectin.^22^ Quantitative RT-PCR was performed and the levels of *Ctgf, Collagen I, Collagen III,* and *Fibronectin* were found to be increased in the *Eva1a*-deficient group (Figures 4c–f). Thus, the results of these experiments suggest that Eva1a expression in mice attenuates cardiac fibrosis.

### Loss of *Eva1a* decreases autophagy and increases apoptosis in heart tissue

**Figure 5:**
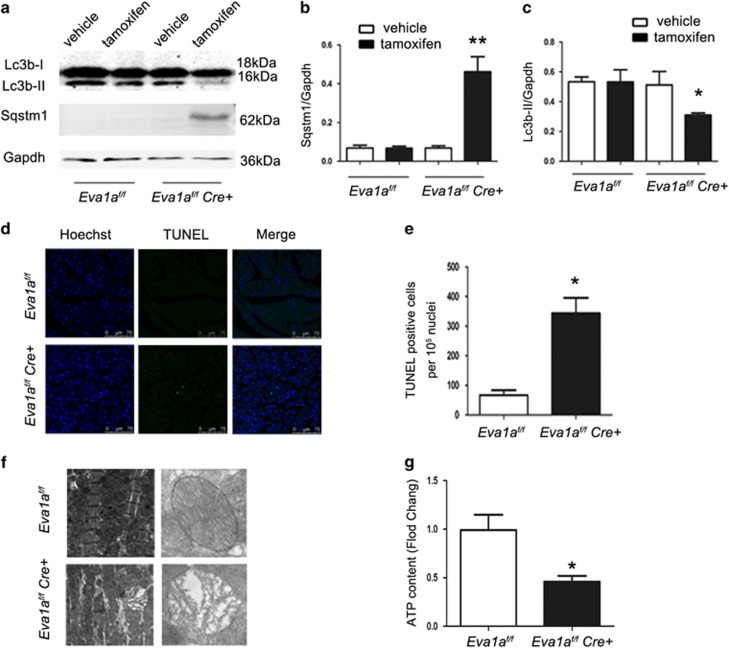
Assessment of autophagy and apoptosis in *Eva1a*-deficient mice. (**a**) Representative western blot analysis of Lc3b and Sqstm1 in heart extracts obtained from different groups. (**b** and **c**) Densitometric analysis of Sqstm1 and Lc3b-II (*P<0.05, **P<0.01, *n*=4). (**d**) Representative images of TUNEL staining (green) and Hoechst staining (blue) of nuclei in cryosectioned heart tissue. (**e**) Quantification of positive cells displaying terminal deoxynucleotidyl transferase-mediated dUDP nick-end labelling (TUNEL) staining. (**f**) Ultrastructural images reveal the presence of swollen mitochondria and lipid accumulation in *Eva1a*-deficient mice. (**g**) Detection of ATP levels (*P<0.05, *n*=3)

Previous studies have shown that autophagy increases protein turnover during cardiac remodelling.^23^ Western blot analysis was carried out to study the expression levels of two autophagy-associated markers, Lc3b and Sqstm1. The results of the western blot analysis showed that the Lc3b-II/Gapdh level was significantly lower in the *Eva1a*-deficiency group than in the other groups (Figures 5a and c), whereas the Lc3b-II/Gapdh level in the other groups were not significantly different (Figures 5a and c). It has been widely recognized that the Sqstm1 protein serves as a link between Lc3 and ubiquitinated substrates. It incorporates into the completed autophagosome and is degraded in lysosomes. Therefore, the Sqstm1 level can serve as a marker for autophagic flux. Our results showed that *Sqstm1* expression was lower at the baseline than after tamoxifen treatment, and there were significant differences in the expression levels between the vehicle- and tamoxifen-treated groups (Figures 5a and b). Consistent with this result, more ubiquitinated inclusions accumulated in the heart tissue in *Eva1a*-deficiency group (Supplementary Figures S1a). Furthermore, Nbr1, another well-characterized autophagy substrate, also dramatically elevated in the *Eva1a*-deleted heart tissue, but not in the wild-type heart tissue (Supplementary Figures S4). These results indicate that loss of Eva1a leads to a significant decrease of autophagic flux.

As apoptosis and autophagy have simultaneously been observed in the failing human heart and as EVA1A is known for its important role in apoptosis regulation,^18, 20^ we asked whether cardiac remodelling is associated with apoptosis. To answer this question, we performed a terminal deoxynucleotidyl transferase-mediated dUDP nick-end labelling (TUNEL) assay using heart sections from all four groups. Results of the TUNEL assay revealed apoptotic cells in all the mice groups. The *Eva1a*-deficient mice had a significantly higher proportion of apoptotic cells than other mice (Figures 5d and e). We also examined cleaved caspase-3, an activated form of caspase-3 that acts as a lethal protease at the most distal stage of the apoptosis pathway. Our results revealed that the cleaved caspase-3 signalling was significantly higher in *Eva1a*-deficient mice than in other mice (Supplementary Figures S1b). However, there was no significant difference in the proportion of apoptotic cells between the vehicle-treated groups (data not shown). These data suggest that apoptosis may have exacerbated cardiac remodelling in *Eva1a*-deficient mice.

### Loss of *Eva1a* in heart affects energy metabolism

Transmission electron microscopy (TEM) of *Eva1a*-deficient hearts showed disorganized sarcomere structure, mitochondrial misalignment, and aggregation (Figure 5f). As the heart is rich in mitochondria, which is closely related to energy metabolism, we investigated whether deficiency of *Eva1a* led to the lack of ATP generation. An ATP kit was used to detect the ATP content in the heart, and the results revealed a significantly lower ATP content in *Eva1a*-deficient mice than in other mice (Figure 5g), suggesting that Eva1a may have a role in mitochondrial energy metabolism and regulation of cardiac remodelling.

Owing to the disorganized mitochondria observed in *Eva1a*-deficient mice hearts, we studied some of the mitochondria-related proteins. The levels of Drp1, which mediates mitochondrial division,^24^ were also downregulated in *Eva1a*-deficient mice (Supplementary Figures S2a and b). Tomm20, which is a mitochondrial marker,^25^ was accumulated in the *Eva1a* knockout group (Supplementary Figures S2a and c). The other proteins detected, including Pink1, Parkin, Bnip3, mitofusin2, and Pgc1, revealed no obvious changes (Supplementary Figures S2a). Taken together, our results suggested that loss of *Eva1a* led to the inhibition of autophagy, resulting in the accumulation of some impaired mitochondria in the hearts of *Eva1a* knockout mice.

### Eva1a modulates autophagy through the LKB1/AMPK-Mtor pathway

**Figure 6:**
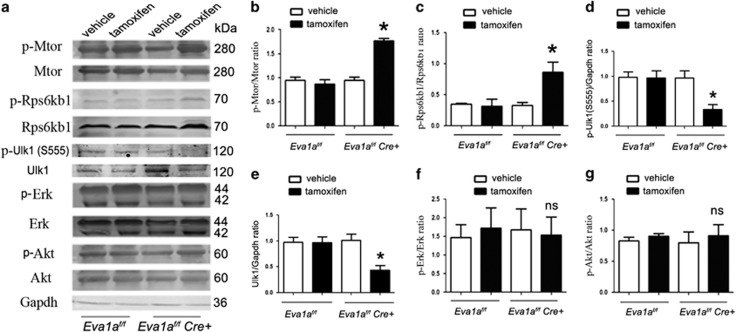
*Eva1a* deficiency increases the Mtor signalling pathway. (**a**) Representative western blot analysis of Mtor, Rps6kb1, Ulk1, Erk, and Akt, and their phosphorylated forms in heart extracts obtained from different groups of mice. (**b**–**g**) Densitometric analysis of p-Mtor, p-Rps6kb1, p-Erk, and p-Akt (*P<0.05, *n*=3)

We then investigated the mechanisms of the defective autophagy in *Eva1a*-deletion mice. The mammalian target of rapamycin (Mtor) has been known as a negative regulator of autophagy. The results showed that the phosphorylation levels of Mtor at Ser2448 (activation site) and Rps6kb1 (70-kDa ribosomal protein S6 kinase, polypeptide 1), as well as Eif4ebp1 (eukaryotic translation initiation factor 4E binding protein 1), two downstream effectors of Mtor were elevated in *Eva1a* knockout hearts (Figures 6a–c, Supplementary Figure S3a and b). In addition, the levels of total and phosphorylated Ulk1 at Ser555 (activation site)^26^ were both decreased in *Eva1a*-deficient mouse hearts (Figures 6a, d, and e). These data implied that the defective autophagy in *Eva1a* knockout mice might be related to Mtor activation and Ulk1 downregulation.

The main regulatory molecules upstream of the Mtor signalling pathway include PIK3CA/AKT, MAPK/ERK, and LKB1/AMPK.^18, 27, 28, 29^ Therefore, we evaluated the phosphorylation levels of Akt, Erk, Lkb1, and Ampk, and the results revealed that the phosphorylation levels of Akt and Erk in heart tissue did not differ between the *Eva1a* knockout mice and other mice (Figures 6a, f, and g), but *Eva1a* deletion reduced the phosphorylation levels of Lkb1 and Ampk (Supplementary Figures S3c–e).

To investigate the significant involvement of Mtor and autophagy in the development of the cardiac phenotype, we used the Mtor-specific inhibitor rapamycin for the rescue experiments. The results indicate that rapamycin could partially rescue left ventricular function by increasing the EF in the *Eva1a*-deficiency group, and reduces the level of the hypertrophic markers Anp and Bnp, suggesting that the cardiac dysfunction induced by *Eva1a* KO is mediated by Mtor activation (data not shown). Taken together, these results suggested that loss of Eva1a leads to the inhibition of the Lkb1–Ampk axis, resulting in the activation of Mtor signalling and decreased autophagy in the *Eva1a* knockout hearts.

## Discussion

In the present study, we investigated the role of *Eva1a* in cardiac remodelling using an *Eva1a* knockout mice model. We demonstrated that Eva1a attenuated cardiac remodelling by modulating autophagy. Genetic disruption of *Eva1a* disrupted the autophagic machinery, consequently impairing cardiac homeostasis, leading to mitochondrial damage, and decreasing the ATP content, and the clearance of impaired mitochondria. This effect may be associated with the decreased autophagy via the Lkb1/Ampk-Mtor signalling pathway. The results of our study suggested that *Eva1a* may be a promising therapeutic candidate for heart disease.

Heart failure has become one of the most serious threats to human health worldwide. Cardiac remodelling, which is triggered by a variety of factors, is a factor initiating heart failure. Therefore, exploring the molecular mechanisms underlying cardiac remodelling can shed insights into the clinical prevention and treatment of heart failure. Autophagy is a complex metabolic programme that is closely related to many diseases, and it has an important role in the regulation of cardiovascular system.

Eva1a is a novel membrane protein that is an important autophagy- and apoptosis-related molecule. Several previous studies have demonstrated the association of this protein with many pathological conditions and diseases.^18, 19, 20, 30^ However, the role of Eva1a in cardiac remodelling remains unknown. Our results demonstrated moderate *Eva1a* mRNA expression in the adult heart tissue. In addition, we first examined gene expression in heart extracts from adult mice subjected to ISO treatment for 2 weeks. There was a significant increase in the *Eva1a* expression in ISO-induced hypertrophied hearts. These data suggest that Eva1a is potentially involved in cardiac remodelling.

In this study, we examined the cardiac structure and function of mice using small-animal ultrasound equipment. Our results indicated that the EF and FS were significantly lower in the *Eva1a*-deficient mice on the third day after injection of tamoxifen and the LVID-d was significantly increased; however, the LVPW-d showed no marked difference between the groups. The results of the above suggest that the *Eva1a*-deficient mice showed a reduction in cardiac function and cardiac centrifugal expansion. These observations are supported by similar findings in *Atg5*- and *MCL-1*-deficient genetic models.^15, 30, 31^ We developed an *Eva1a* knockout mice model, and *Eva1a* was systemically knocked out in *Eva1a*^*−/–*^ mice during embryonic development. We found no significant alterations in the birth and survival rate, and no obvious abnormalities in cardiac function in adult mice (data not shown). We hypothesized that the different phenotypes of systemic *Eva1a* knockout mice and adult inducible cardiac-specific *Eva1a*-deficient mice may be due to the embryonic stage of development-related genes compensation, which is similar to the results reported in some international studies.^9, 15^ Moreover, no international studies have investigated the role of Eva1a in cardiac function, and our study demonstrates that Eva1a has a protective effect on cardiac function at baseline.

Pathological changes are of unique significance in the diagnosis of myocardial hypertrophy. Histomorphological characteristics can reflect the extent of myocardial damage; furthermore, they can be studied to determine the curative effect of therapy. Our study showed that the heart weight/body weight of *Eva1a*-deficient mice showed a significant increase compared with the other groups. Haematoxylin and eosin (H&E) staining of *Eva1a*-deficient heart tissue revealed disorganized cardiomyocytes, cardiac hypertrophy, and swollen cytoplasm. The cross-sectional area of cardiomyocytes was significantly higher in *Eva1a*-deficient mice compared with that in the other mice groups. Currently, the embryonic genes *Anp* and *Bnp* have been used as important cardiac hypertrophy markers. Compared with other groups, *Anp* and *Bnp* were markedly increased in *Eva1a*-deficient mice; however, there was no significant difference in the *β*-Mhc expression between the four groups. This is the first study to discover that *Eva1a* deficiency can cause myocardial hypertrophy, indicating the important role that Eva1a had in maintaining cardiac homeostasis.

Structural changes in cardiac cells are another important manifestation of cardiac fibrosis, and it occurs as proliferation of a large number of fibroblasts and deposition of collagen fibre. Sirius red staining may reflect the interstitial remodelling in the myocardium, which mainly occurs for the reconstruction of the collagen fibre network; therefore, it is often used to detect the degree of cardiac fibrosis. In our study, none of the mice exhibited myocardial fibrosis on the third day (data not shown); however, partial *Eva1a-*deficient mice exhibited obvious cardiac fibrosis at 2 weeks. This may be due to the gradual development of fibrosis. On day 3 after tamoxifen induction, cardiac fibrosis in *Eva1a*-deficient mice may be too acute for compensatory mechanisms to be effective. Meanwhile, the results of the ultrasound analysis indicated that the cardiac function of *Eva1a*-deficient mice had improved at 2 weeks compared with that on day 3. We consider that myocardial fibrosis may be caused by a compensatory response in the heart. On the other hand, oxidative and subsequent nitrosative damage of the myocardium and vasculature have been described as major primary mechanisms leading to pathological alterations associated with many cardiovascular diseases. Autophagy and oxidative stress are closely related in cardiomyopathy; the fibrosis also may be due to increased oxidative stress.^32^ A more detailed mechanism is still in progress.

Cardiomyocytes are widely used in the research of cardiac autophagy at the baseline. Autophagy can be a protective mechanism in cardiomyocytes under normal or mild levels of stress. As damage to the mitochondria could release proapoptotic factors, autophagy can prevent apoptosis activity and consequently inhibit myocardial apoptosis. Thus, it is believed that the protective effect of autophagy is related to its inhibition of apoptosis; and by providing amino acids, fatty acids, and other substances. Moreover, autophagy has a role in maintaining the cytoplasm and quantity, and degrades damaged organelles and proteins to provide ATP.

Previous studies have shown that autophagy increases protein turnover during cardiac remodelling.^23^ Therefore, we examined the expression of two autophagy markers, Lc3b and Sqstm1. The Lc3b-II/Gapdh level was significantly decreased and the Sqstm1 expression level was significantly increased in *Eva1a*-deficient mice compared with the other mice, indicating that the autophagy in myocardial tissue was decreased in *Eva1a*-deficient mice. Collectively, these data suggest that *Eva1a* may have a beneficial role in the heart via autophagy. Abnormal autophagy could lead to cell death.^9, 30^

The results of our study revealed a significantly higher proportion of apoptotic cells in the myocardial tissue of *Eva1a*-deficient mice than in the other groups, indicating that abnormal autophagy that occurs after the loss of *Eva1a*, which eventually leads to increased apoptosis, may be one of the reasons for the cardiac remodelling.

The autophagy-initiating factor ULK1, which belongs to the serine/threonine kinase family, is required for autophagy induction.^27, 33^ ULK1 forms a complex with Atg13 and FIP200 to regulate the initial step of autophagy induction in mammalian cells. In addition, Ampk-dependent phosphorylation of Ulk1 (S555) is critical for translocation of Ulk1 to mitochondria and promotion of mitophagy. Considering the disorganized mitochondria in Eva1a-deficient mice hearts, the levels of total Ulk1 and phosphorylated Ulk1 at Ser555 (activation site) are both decreased in Eva1a-deficient mouse hearts (Figures 6a, e and f). Drp1 is located in the mitochondrial outer membrane and mediates mitochondrial division. In *Drp1*-deleted cardiomyocytes, mitochondria exhibited increased connectivity, accumulated ubiquitinated proteins, and decreased respiration,^25^ which is similar to that observed in *Eva1a*-deleted mice. This suggests that *Eva1a* deletion may inhibit mitophagy by downregulating Drp1 expression in some degree. The inhibition of mitophagy lead to damaged mitochondria that cannot be degraded and accumulation of the mitochondrial marker Tomm20.

There is a close relationship between autophagy and energy metabolism. In the normal heart, mitochondrial autophagy occurs to maintain mitochondrial function, promote the generation of mitochondria, and to increase the generation of energy. In addition, autophagy degrades long-lived proteins, producing amino acids that generate ATP in the tricarboxylic acid cycle. We wondered whether the decreased autophagy in *Eva1a* knockout hearts could affect the normal function of mitochondria, resulting in energy metabolism disorders, and consequently influence cardiac remodelling. The results of the electron microscopic analysis demonstrated morphological abnormalities, disordered arrangement, and swelling in the mitochondria of *Eva1a*-deficient hearts; moreover, the mitochondria appeared blurred due to lack of particles in the electron-lucent matrix. These aberrant concentric membranous structures were similar to those observed in *Atg5*-deficient hearts.^9^ Finally, as the heart is rich in mitochondria, which are important organelles in energy metabolism, we investigated whether deficiency of *Eva1a* affected ATP generation. The ATP content was significantly lower in *Eva1a*-deficient mice than in the other mice, indicating that normal mitochondrial function and mitochondrial energy metabolism may be affected in *Eva1a*-deficient mice by decreased autophagy, which may be one of the important reasons for the accelerated cardiac remodelling.

In summary, the present study demonstrates that Eva1a protects against cardiac remodelling and heart failure. The protective effects of Eva1a appear to involve autophagy via inhibition of the Mtor signalling pathway. Future studies should be conducted to explore the potential for Eva1a as a therapeutic target in heart failure.

## Materials and methods

### Generation of tissue-specific *Eva1a*-deficient mice

*Eva1a*^*flox/flox*^ mice with a C57BL/6 background were constructed by the Chinese Academy of Medical Sciences and bred at the Experimental Animal Center, Peking University Health Sciences Center (Beijing, China). The animal experimental protocol was approved by the Biomedical Research Ethics Committee of Peking University (LA 2010-048) and strictly adhered to the American Physiological Society's Guiding Principles in the Care and Use of Vertebrate Animals in Research and Training. Eight-week-old male C57BL/6 mice were provided by the Animal Department of Peking University Health Science Center (Beijing, China). The mice were housed in groups of four in a room with controlled temperature (25±2 °C), with free access to food and water. All the animal experiments were approved by Biomedical Research Ethics Committee of Peking University.

Mice harbouring a homozygous conditional null mutation in *Eva1a*^*flox/flox*^ were crossed with transgenic *α-MHC MerCreMer* mice, which expressed a tamoxifen-inducible *Cre* recombinase (*MerCreMer*) under the transcriptional control of the cardiomyocyte-specific *α-MHC* promoter, to generate conditional cardiomyocyte-specific *Eva1a* knockout mice. All the mice had a C57BL/6 background, and *Eva1a*^*flox/flox*^ littermates without *MerCreMer* were used as the controls for this study. To selectively delete *Eva1a* in cardiomyocytes, we treated 8-week-old *Eva1a*^*flox/flox*^*: α-MHC MerCreMer*^*+*^ (*Eva1a*^*f/f*^*Cre*^*+*^) and *Eva1a*^*flox/flox*^ *MerCreMer*^*−*^ (*Eva1a*^*f/f*^) mice with 80 mg/kg tamoxifen once a day for 3 days and analysed the mice. This dose resulted in the most rapid and effective knockdown of the *Eva1a* gene without any cardiotoxicity.

### RNA isolation and real-time RT-PCR

Total RNA was prepared from mice tissues using TRIZOL reagent (15596, Invitrogen, Carlsbad, CA, USA), and cDNA was synthesized using Revert Aid First Strand cDNA Synthesis Kit (K1622, Thermo Scientific, Waltham, MA, USA). The expression of mRNA was analysed by quantitative real-time RT-PCR (Applied Biosystems, Waltham, MA, USA StepOne Plus) and normalized to the expression of the *Gapdh* housekeeping gene. Real-time RT-PCR was performed in triplicate for each sample. Table 1 presents a list of the primers used for real-time PCR.

### Southern blot analysis

Genomic DNA from heart tissue was separated and purified by DNeasy Tissue kit (69504, Qiagen, Hilden, Germany). Southern blot analysis was performed according to the standard procedure. The isolated DNA was digested by *Eco*RV enzyme. The labelled probe was designed at the third exon using the forward primer 5′-TCTAAAGGACTCCGTGAA-3′ and the reverse primer 5′-ACCTCTGGCTTCCATTCT-3′.

### Western blot analysis

Briefly, the mice organs were collected and disrupted in lysis buffer containing protease inhibitors (Roche Diagnostics, Berlin, Germany). After centrifugation, the supernatant was collected, and equivalent amounts of protein were subjected to sodium dodecyl sulphate-polyacrylamide gel electrophoresis (SDS-PAGE) and transferred to a nitrocellulose membrane. The protein bands were visualized using DyLight 800/DyLight 680-conjugated secondary antibodies, and an infrared fluorescence image was obtained using an Odyssey infrared imaging system (LI-COR Biosciences, Lincoln, NE, USA). Western blot analyses were performed by ImageJ with anti-Gapdh (KM9002, Sungene, Tianjin, China), anti-Lc3b (SAB4200361, Sigma, St Louis, MO, USA), anti-Sqstm1 (PM045, MBL International, Japan), and anti-Eva1a (NB110-74787, Novusbio, Littleton, CO, USA) antibodies. Antibodies against Ulk1, Akt, Mtor, Erk1/2, Lkb1, Ampk, Rps6kb1, and Eif4ebp1 and against phosphorylated Ulk1, Akt, Mtor, Erk1/2, Lkb1, Ampk, Rps6kb1, and Eif4ebp1 were purchased from Cell Signaling Technology (Boston, MA, USA). Antibodies against Drp1, Tomm20, Pink1, Parkin, Bnip3, Mitofusin2, and Pgc1 were purchased from Abcam (Cambridge, UK). DyLight 800/DyLight 680-conjugated secondary antibodies against mouse or rabbit IgG were purchased from Rockland Immunochemicals (Limerick, PA, USA).

### Transmission electron microscopy

Cardiac tissue was initially fixed in 0.1 M sodium phosphate buffer containing 3% glutaraldehyde (pH 7.4) and then fixed in 0.1 M sodium phosphate buffer containing 1% OsO_4_ (pH 7.2) for 2 h at 4 °C. The tissue was dehydrated in a graded ethanol series. Then the tissue was embedded in Ultracut (Leica Ultracut) and sliced into 60 nm sections. The ultrathin sections were stained with uranyl acetate and lead citrate, and observed under a JEM-1230 transmission electron microscope.

### Echocardiographic analysis

Mice were anaesthetized with 1% isoflurane (Baxter Healthcare Corporation, New Providence, NJ, USA). Echocardiographic images were obtained using a Visualsonics high-resolution Vevo770 system (VisualSonics Inc., Toronto, ON, Canada). Two-dimensional parasternal long-axis and short-axis views were obtained at the level of the papillary muscle. Diastolic left ventricular posterior wall thickness (LVPW-d) and systolic left ventricular posterior wall thickness (LVPW-s) were measured to calculate the EF and FS. All the measurements were averaged from three consecutive cardiac cycles.

### Quantitative histological analyses

After the mice were killed, the hearts were harvested and perfused in retrograde with cold phosphate-buffered saline (PBS), fixed with 4% paraformaldehyde for 8 h, dehydrated in 20% sucrose for 24 h, and embedded in paraffin. Serial 5 *μ*m thick sections were stained with H&E for morphological analysis, or picrosirius red for the detection of fibrosis. For morphometric analysis, photographs of left ventricular sections cut from the same location of each heart were observed under × 400 magnification (Leica Microsystems Imaging Solutions Ltd., Cambridge, UK). Interstitial fibrosis was visualized with picrosirius red staining, and the cardiac fibrosis volume fraction was calculated as the ratio of the stained fibrotic area to the total myocardial area. For TUNEL staining, heart tissues were embedded in a freezing matrix. Serial 7 mm cryostat sections were prepared and stored at −20 °C until use. TUNEL assays were performed with the *in situ* cell death detection kit (Roche Applied Science, Indianapolis, IN, USA), according to the manufacturer's instructions. The sections were counterstained with 4', 6-diamidino-2-phenylindole (DAPI).

### Detection of ATP levels

The ATP levels of heart tissue from mice were measured using a firefly luciferase-based ATP assay kit (Beyotime, Shanghai, China), according to the manufacturer's instructions. After the indicated treatments, cardiomyocytes were lysed and centrifuged at 12 000 × *g* for 5 min. Supernatants (100 *μ*l) were mixed with 100 *μ*l of ATP detection working dilution in a white 96-well plate. Standard curves were also generated, and the protein concentration of each treatment group was determined using the Bradford protein assay. The total ATP levels were expressed as nmol/mg protein. This experiment was repeated three times.

### Statistical analysis

The data are summarized as means±S.E.M. Differences between groups were compared using Prism 5 (GraphPad Software Incorporate, La Jolla, CA, USA) with unpaired two-tailed Student's *t*-test. A *P*-value <0.05 was considered statistically significant.

## Figures and Tables

**Table 1:** Primers used in this study

*Gene*	*5′-primer*	*3′-primer*
*Gapdh*	CAAGGTCATCCATGACAACTTTG	GTCCACCACCCTGTTGCTGTAG
*Eva1a*	AGACAACCTTTTCCTCCCAC	AGAGACAAAGTACAGAGCGGC
*Anp*	GTACAGTGCGGTGTCCAACA	TCTCCTCCAGGTGGTCTAGCA
*Bnp*	CACCGCTGGGAGGTCACT	GTGAGGCCTTGGTCCTTCAA
*β-Mhc*	GCATTCTCCTGCTGTTTCCTT	TGGATTCTCAAACGTGTCTAGTGA
*Ctgf*	TGACCTGGAGGAAAACATTAAGA	AGCCCTGTATGTCTTCACACTG
*Fibronectin*	CACGGAGGCCACCATTACT	CTTCAGGGCAATGACGTAGAT
*Collagen I*	CTCCTGGCAAGAATGGAGAT	AATCCACGAGCACCCTGA
*Collagen III*	AACAGAGGTGAAAGAGGATCTGA	TCACCTCCAACTCCAGCAAT
