# Supplementary material for: Knockout of Eva1a leads to rapid development of heart failure by impairing autophagy
Source: Cell Death Dis. 2017 Feb 2;8(2):e2586–. doi: 10.1038/cddis.2017.17 (PMC5386466; doi:10.1038/cddis.2017.17)
Supplement: Supplementary Information [file cddis201717x1.doc]

**Supplementary data**

***Knockout of Eva1a leads to rapid development of heart failure by impairing autophagy***

Shu Zhang ^1,†^, Xin Lin ^2,3,†^, Ge Li ^2,3^, Xue Shen ^4^, Di Niu ^4^, Guang Lu ^4,5^, Xin Fu^1^, Yingyu Chen ^2,3^, Ming Cui ^1,*^, Yun Bai ^4,*^

^1^ Department of Cardiology, Peking University Third Hospital, Beijing 100191, China

^2^ Department of Immunology, School of Basic Medical Sciences, Peking University Health Science Center, Beijing 100191, China

^3^ Peking University Center for Human Disease Genomics, Beijing 100191, China

^4^ Department of Cell Biology, School of Basic Medical Sciences, Peking University Health Science Center, Beijing 100191, China

^5^ Department of Physiology, Yong Loo Lin School of Medicine, National University of Singapore, Singapore 117597

† These authors contributed equally to this work and share the first authorship.

* Corresponding authors. E-mail addresses: baiyun@bjmu.edu.cn (Y. Bai) or mingcui@bjmu.edu.cn (M. Cui)

Phone: +86-10-82801495 (Y. Bai) or +86-13021017585 (M. Cui).

**Materials and Methods**

**Immunohistochemistry (IHC) staining**

The sections were deparaffinized and rehydrated. Antigen retrieval was performed in a pressure cooker at 100°C for 2 minutes in 0.01M sodium citrate (pH 6.0), and endogenous peroxidase activity was blocked with 3% hydrogen peroxide. The slides were then blocked with goat serum and incubated with the anti-Ubiquitin (P4D1) Mouse monoclonal antibody or anti-Cleaved Caspase-3 (Asp175)(5A1E) Rabbit monoclonal antibody(purchased from Cell Signaling Technology) at 4°C overnight. After washing 3 times in PBS, the sections were conducted with DAB Detection Kit (PV-6000-D, Origene, China) according to the manufacturer’s instructions. The sections were developed with DAB substrate (PV-6000-D, Origene, China) and counter-stained with hematoxylin. Samples were then dehydrated and sealed with coverslips. Negative controls were performed by replacing the primary antibody with rabbit IgG.

**Supplementary Figure Legends**

Supplementary Figure S1*. Eva1a* deficiency increases the accumulation of ubiquitin and the levels of cleaved caspase3. (a and b) Representative images of immunohistochemical staining of ubiquitin and cleaved caspase3 in heart sections. Scale bar = 50 µm.

Supplementary Figure S2. Eva1a deficiency regulates mitophagy signalling pathway. (a) Representative Western blot analysis of Drp1, Tomm20, Pink1, Parkin, Bnip3, Mitofusin2, and Pgc1 in heart extracts obtained from different groups of mice. (b and c) Densitometric analysis of Drp1 and Tomm20 (*p < 0.05, n = 3).

Supplementary Figure S3. *Eva1a* deficiency decreases 4Ebp1 and promotes the Lkb1-Ampk signalling. (a) Representative Western blot analysis of p-4Ebp1 (T37/46) and 4Ebp1 in heart extracts obtained from different groups of mice. (b) Statistical analysis of p-4Ebp1 (**p* < 0.05, n = 3). (c) Representative Western blot analysis of Lkb1, Ampkα and their phosphorylated forms in heart extracts obtained from different groups of mice. (d and e) Statistical analysis of p-Lkb1, p-Ampkα (**p* < 0.05, n = 3).

Supplementary Figure S4. *Eva1a* deficiency increases the levels of Nbr1. (a and b) Representative images of immunohistochemical staining of Nbr1 in heart sections. Scale bar = 50 µm.
