# Supplementary figures and images for: Knockout of Eva1a leads to rapid development of heart failure by impairing autophagy
Source: Cell Death Dis. 2017 Feb 2;8(2):e2586–. doi: 10.1038/cddis.2017.17 (PMC5386466; doi:10.1038/cddis.2017.17)

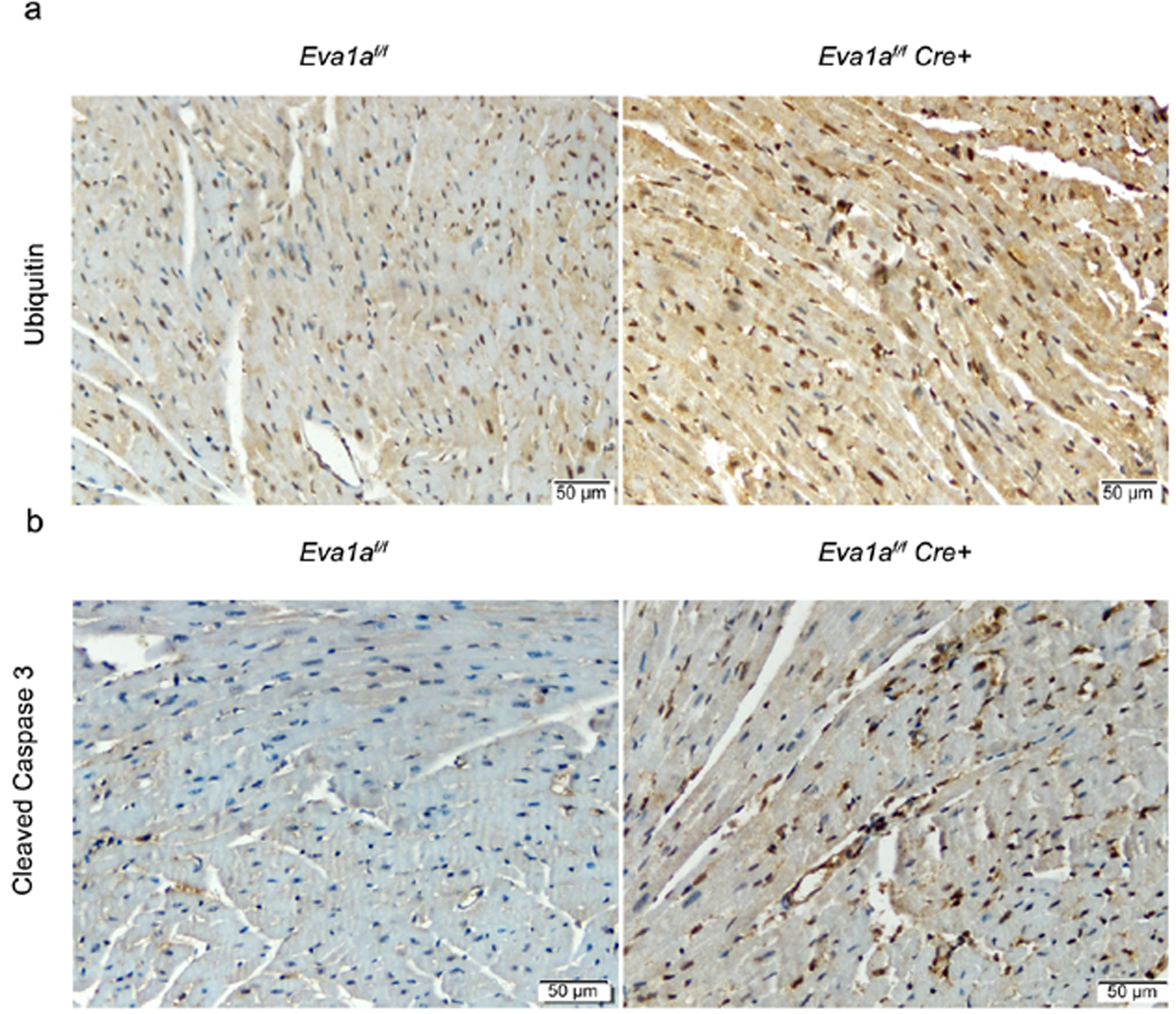

Supplement: Supplementary Figure S1 [file cddis201717x2.tif]

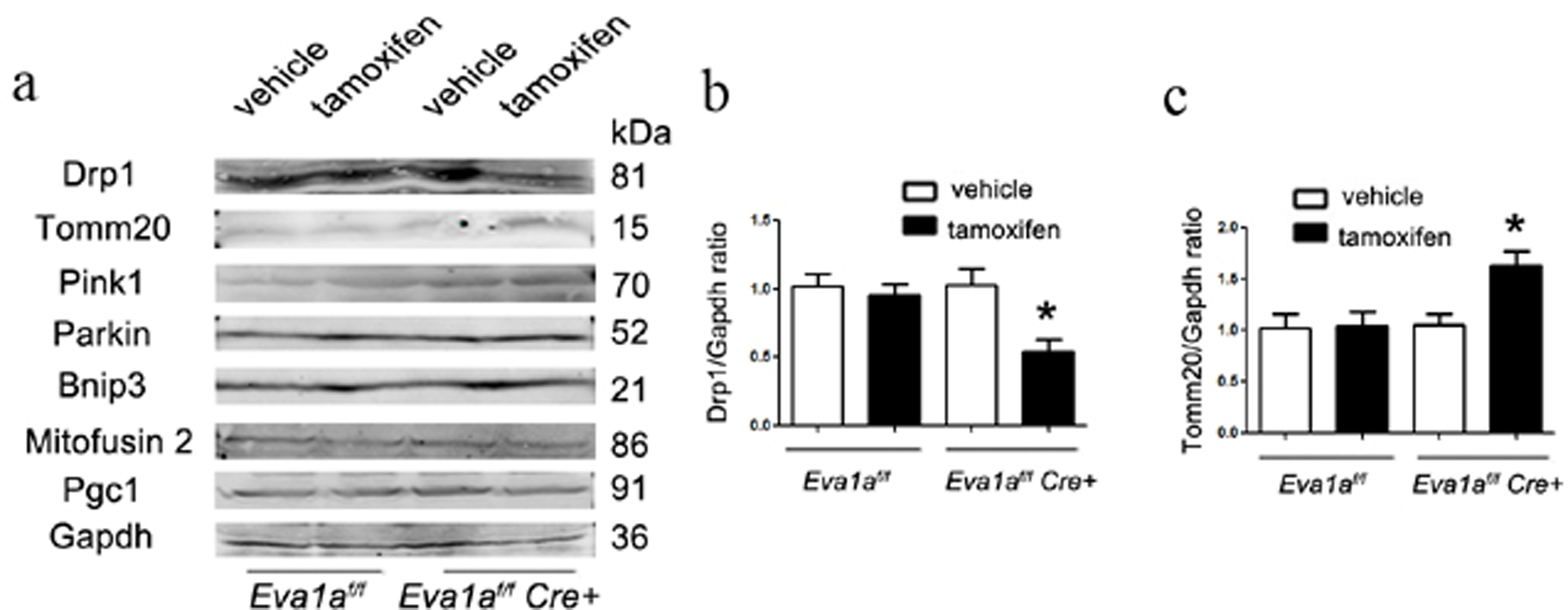

Supplement: Supplementary Figure S2 [file cddis201717x3.tif]

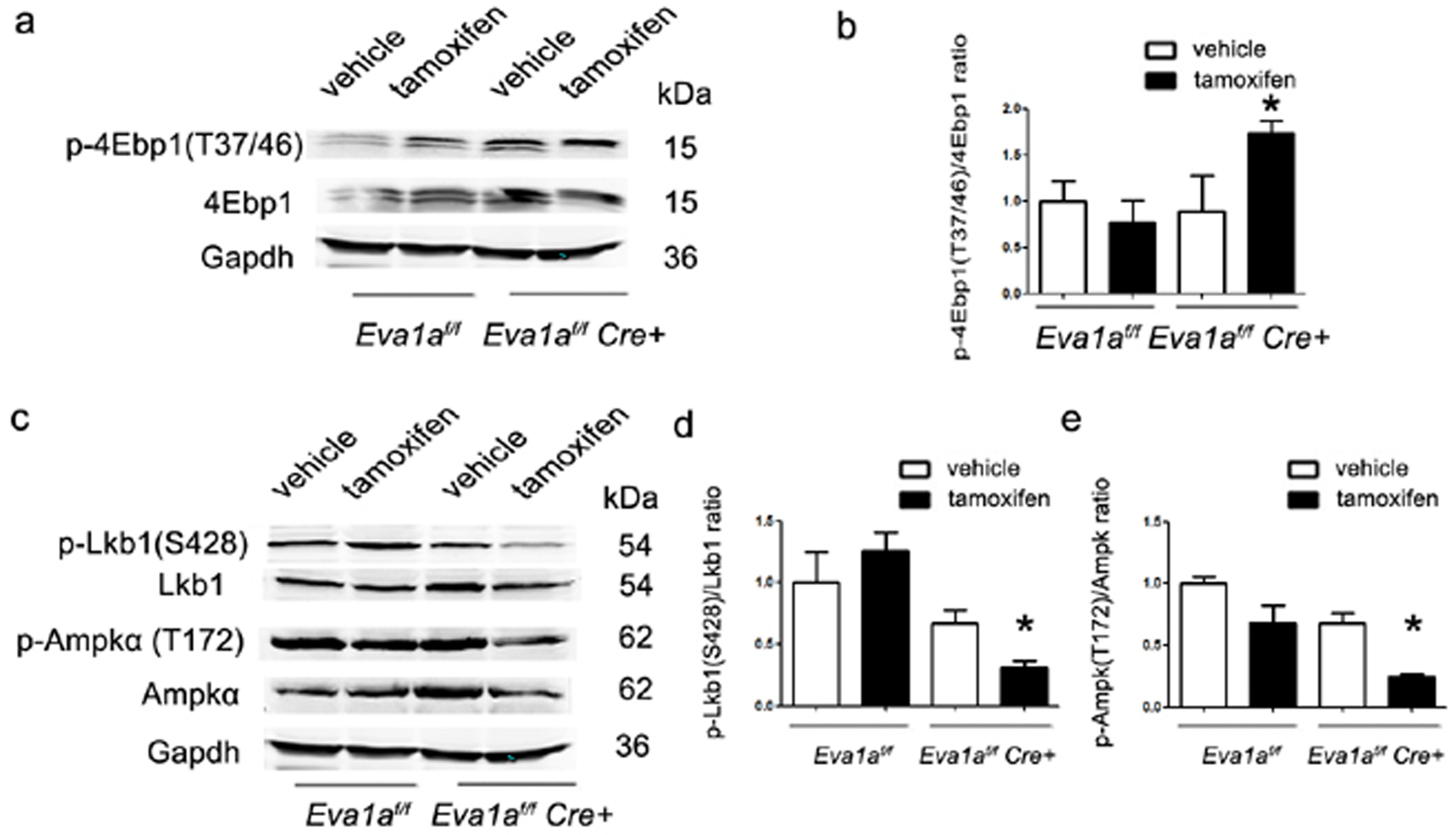

Supplement: Supplementary Figure S3 [file cddis201717x4.tif]

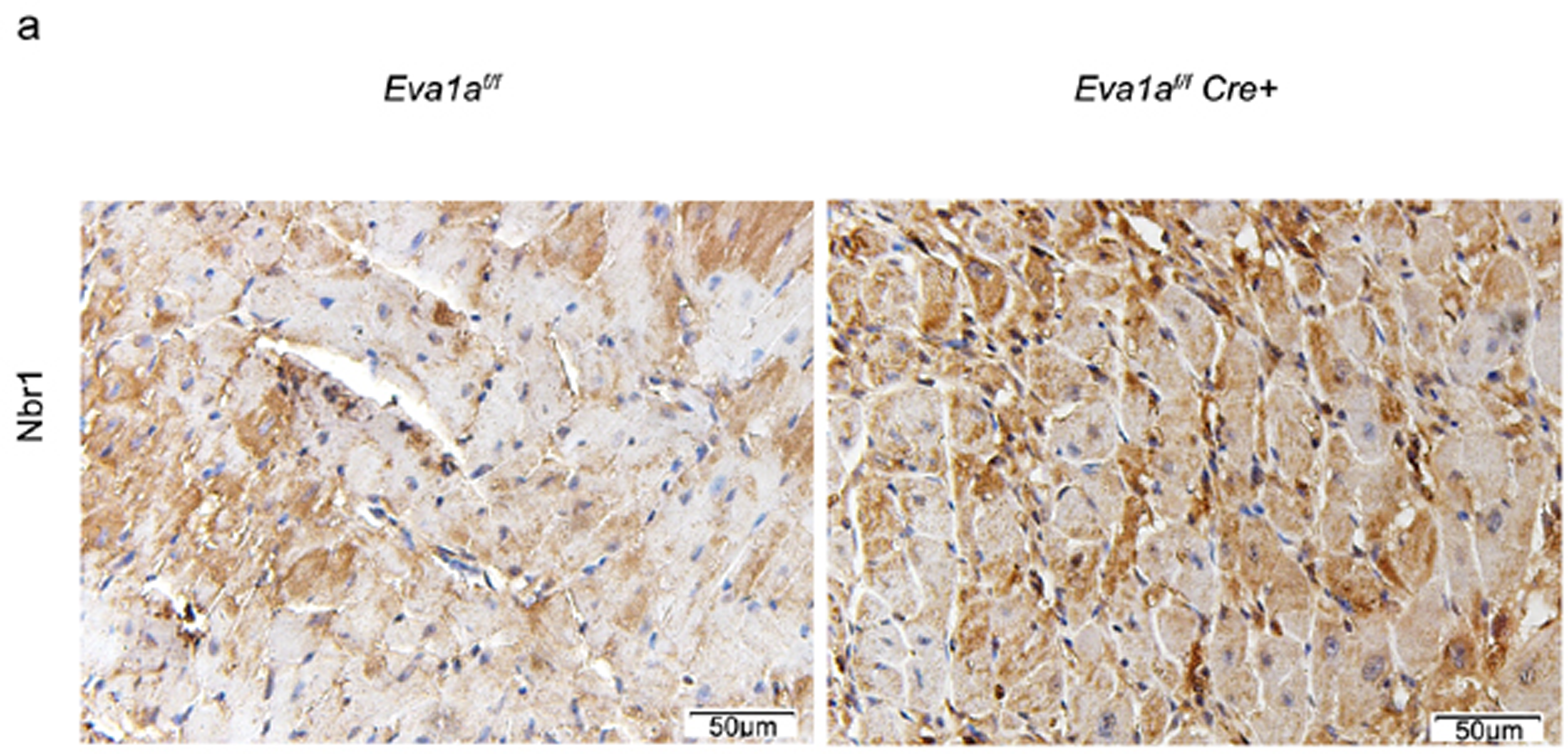

Supplement: Supplementary Figure S4 [file cddis201717x5.tif]
